# Haemodynamic assessment and support in sepsis and septic shock in resource-limited settings

**DOI:** 10.1093/trstmh/try007

**Published:** 2018-02-09

**Authors:** David Misango, Rajyabardhan Pattnaik, Tim Baker, Martin W Dünser, Arjen M Dondorp, Marcus J Schultz, for the Global Intensive Care Working Group, of the European Society of Intensive Care Medicine (ESICM) and the Mahidol Oxford Tropical Medicine Research Unit (MORU) in Bangkok, Thailand

**Affiliations:** 1 Department of Anaesthesiology and Critical Care Medicine, Aga Khan University Hospital, Nairobi, Kenya; 2 Department of Intensive Care Medicine, Ispat General Hospital, Rourkela, Sundargarh, Odisha, India; 3 Department of Anesthesia, Intensive Care and Surgical Services, Karolinska University Hospital, Stockholm, Sweden; 4 Department of Public Health Sciences, Karolinska Institutet; 5 Department of Critical Care, University College of London Hospital, London, UK; 6 Mahidol Oxford Tropical Medicine Research Unit (MORU), Faculty of Tropical Medicine, Mahidol University, 420/6 Rajvithi Road, Bangkok 10400, Thailand; 7 Oxford Centre for Tropical Medicine and Global Health, Nuffield Department of Clinical Medicine, University of Oxford, Oxford, UK; 8 Department of Intensive Care, Academic Medical Center, Amsterdam, The Netherlands

**Keywords:** Circulation, Fluid resuscitation, Inotrope, Sepsis, Septic shock, Vasopressor

## Abstract

**Background:**

Recommendations for haemodynamic assessment and support in sepsis and septic shock in resource-limited settings are largely lacking.

**Methods:**

A task force of six international experts in critical care medicine, all of them members of the Global Intensive Care Working Group of the European Society of Intensive Care Medicine and with extensive bedside experience in resource-limited intensive care units, reviewed the literature and provided recommendations regarding haemodynamic assessment and support, keeping aspects of efficacy and effectiveness, availability and feasibility and affordability and safety in mind.

**Results:**

We suggest using capillary refill time, skin mottling scores and skin temperature gradients; suggest a passive leg raise test to guide fluid resuscitation; recommend crystalloid solutions as the initial fluid of choice; recommend initial fluid resuscitation with 30 ml/kg in the first 3 h, but with extreme caution in settings where there is a lack of mechanical ventilation; recommend against an early start of vasopressors; suggest starting a vasopressor in patients with persistent hypotension after initial fluid resuscitation with at least 30 ml/kg, but earlier when there is lack of vasopressors and mechanical ventilation; recommend using norepinephrine (noradrenaline) as a first-line vasopressor; suggest starting an inotrope with persistence of plasma lactate >2 mmol/L or persistence of skin mottling or prolonged capillary refill time when plasma lactate cannot be measured, and only after initial fluid resuscitation; suggest the use of dobutamine as a first-line inotrope; recommend administering vasopressors through a central venous line and suggest administering vasopressors and inotropes via a central venous line using a syringe or infusion pump when available.

**Conclusion:**

Recommendations for haemodynamic assessment and support in sepsis and septic shock in resource-limited settings have been developed by a task force of six international experts in critical care medicine with extensive practical experience in resource-limited settings.

## Introduction

Recommendations for care in patients with sepsis or septic shock are largely based on evidence originating from resource-rich settings.^1^ It is increasingly appreciated that these recommendations cannot be directly generalized to resource-limited settings for several reasons, including restrictions in human and material resources, but also concerns regarding costs and safety.^2,3^ It is even possible that the efficacy and effectiveness of certain strategies differ between resource-rich and resource-limited settings. Indeed, efficacy and effectiveness could depend on the type of sepsis, and it is well known that non-bacterial sepsis is much more common in resource-limited than in resource-rich settings.^3^

A task force of the Global Intensive Care Working Group of the European Society of Intensive Care Medicine (ESICM) wished to answer five practical questions regarding haemodynamic assessment and support in sepsis and septic shock in resource-limited settings. As recognition of hypoperfusion and return to normal perfusion, as well as detection of fluid responsiveness, could avoid under- or overresuscitation or under- or overuse of vasoactive agents, there is need for affordable bedside tools for tissue perfusion monitoring as well as a better understanding of practicalities of passive leg raise tests in these settings. As costs and the availability of, but also indications for, intravenous fluids can be different in resource-limited settings, certain types and amounts of intravenous fluid should be used during fluid resuscitation, and the proper timing of intravenous fluid treatment for sepsis and septic shock in resource-limited intensive care units (ICUs) is essential. Finally, because of the limited availability of vasopressors and inotropes, and the risks associated with their use, recommendations on their indications, titrations and ways of administration in settings with limited resources are necessary.

Therefore, six international experts in critical care medicine reviewed current guidelines and the existing literature. For this they used the recently updated guidelines of the Surviving Sepsis Campaign^1^ and searched for additional evidence originating from resource-limited settings. They reformulated the existing recommendations for haemodynamic assessment and support, focusing on efficacy and effectiveness and aspects such as availability, feasibility, affordability and safety.

## Methods

Full methods are provided in the supplementary material. The methods followed a similar approach as used previously by other task forces of the Global Intensive Care Working Group of the ESICM.^4–9^ External peer review was provided through the complete panel of the Global Intensive Care Working Group.^3^

### Task force team members

The process for selection of task force members involved in this review and the key issues in haemodynamic assessment and support to be discussed are described in the supplementary material.

### Search strategy

The search strategy for relevant studies was as described for the development of the Surviving Sepsis Campaign: International Guidelines for Management of Sepsis and Septic Shock: 2016 guidelines.^1^ Searched databases included PubMed, MEDLINE, Embase and the Cochrane Libraries, with a focus on investigations originating from resource-limited settings.

### Recommendations

The generated list of recommendations was graded for the level of evidence and strength of each recommendation, using Grading of Recommendations Assessment, Development and Evaluation (GRADE) tools,^10^ details of which are provided in the supplementary material. The primary source of evidence was studies performed in resource-limited settings, and grading of evidence included efficacy and effectiveness, availability and feasibility and affordability and safety in resource-limited settings (detailed in Table 1 in the supplementary material). Recommendations concern adult as well as paediatric populations; where the recommendations were different, these were separated.

Using the principles of GRADE, task force members classified the quality of evidence as high (grade A), moderate (grade B), low (grade C) or very low (grade D) and recommendations as strong (grade 1) or weak (grade 2). The term ‘recommend’ was used for strong recommendations, whereas ‘suggest’ was used in case of lower-level evidence. In case a recommendation was based on expert opinion from the group, it was classified as ‘ungraded’ (UG) (detailed in Table 2 in the supplementary material).

#### Recommendations for simple bedside tools

(1) Which simple bedside tools for assessing tissue perfusion could be useful in sepsis and septic shock in resource-limited settings?

Recommendation: We suggest using capillary refill time, skin mottling scores and, if affordable, skin temperature gradients to assess the adequacy of tissue perfusion in paediatric and adult sepsis and septic shock, either alone or in combination (UG). It remains uncertain whether these tools are effective in malaria. These tools are non-invasive and safe and come at no additional or low cost, although the cost of temperature probes could still be too high for certain resource-limited settings. This recommendation remains weak, mainly because of the absence of evidence that these bedside tools can adequately guide important decisions in haemodynamic support.

##### Rationale

Timely detection of tissue hypoperfusion is one crucial aspect of haemodynamic assessment in patients with sepsis or septic shock. The Surviving Sepsis Campaign does not recommend simple and affordable bedside tools for assessing tissue perfusion.^1^ A search of the literature combining various search terms such as ‘skin perfusion’, ‘skin colour’, and ‘skin temperature gradients’, alone and in combination with diverse search terms covering ‘sepsis and septic shock’ and ‘resource-limited settings’ resulted in 12 articles, the majority still from resource-rich settings.^11–22^

Several studies showed that capillary refill times >5 s following initial haemodynamic optimization are associated with worsening organ failure.^11–13^ Normalization of capillary refill time was prognostic of survival in septic shock patients.^14^ During early septic shock, capillary refill time was found to be a good predictor of short-term mortality^15^ and related to perfusion of the liver, spleen, kidneys and intestines in adults.^16^ There was noticeable variation though in how capillary refill times were checked, at least in investigations involving children (Table 1), and several factors may affect the accuracy of capillary refill time, including ambient temperature and light, the site of measurement and the amount of pressure applied to the capillary bed.^23^ There was debate about whether capillary refill time is subject to interobserver variability.^23,24^ One study in India suggests capillary refill time is insensitive to detect tissue hypoperfusion in patients with malaria.^25^

**Table 1.** Different methods of measuring and interpreting capillary refill time in children

Method	Interpretation
Apply pressure to the nail bed or other area with visible circulation; measure the length of time it takes for blanching to disappear	A capillary refill time <2 s is normal and >4 s is abnormal. A capillary refill time between 2 and 4 s should prompt further consideration of the presence of shock
The preferred location to test capillary refill time is the sternum. If the finger or toe is used, the leg or arm must be elevated. Press firmly for 5 s	A capillary refill time >5 s indicates an inadequate cardiac output
After fingertip pressure to a distal extremity, blood should refill the area in <2 s after release	A capillary refill time >2 s in the setting of other signs of shock indicates a compensated shock state
Press on the sternum or digit at the level of the heart for 5 s	A capillary refill time >2 s is a clinical feature of shock
Cutaneous pressure on the sternum or on a digit for 5 s	A refill time >2 s can indicate poor skin perfusion, a sign that may be helpful in early septic shock
Grasp the child’s thumb or big toe between finger and thumb and look at the pink of the nail bed. Apply minimal pressure necessary for 3 s to produce blanching of the nail bed. The time to capillary refill is from the moment of release until a total return of the pink colour	Capillary refill time should be <3 s. If >3 s the child may have a problem with shock

Adapted and modified from Pandey and John.^44^

**Figure 1.**
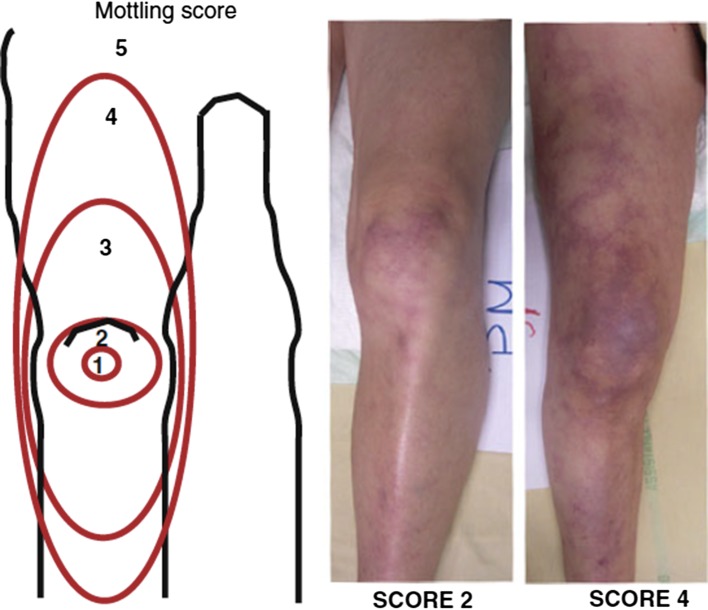
Skin mottling score. Adapted from Ait-Oufella et al.^17^

Mottling, patchy skin discolorations due to heterogenic small vessel vasoconstriction that usually start around the knees and elbows in patients with shock could also reflect abnormal skin perfusion. A score that is simple to apply at the bedside, using a scale from 0 (‘no mottling’) to 5 (‘grave mottling’) (Table 2 and Figure 1), related well to plasma lactate levels, urine output, degree of organ dysfunction and even mortality in patients with septic shock.^17^ Patients whose mottling score decreased during the resuscitation period had a better prognosis.^17^ The prognostic value of this score was confirmed in other cohorts of critically ill patients.^18,19^ The mottling score had good reproducibility and did not suffer from interobserver variability.^17^

**Table 2.** Skin mottling score after initial fluid resuscitation

Score		Description
0	No	No mottling
1	Modest	Coin size, localized to the centre of the knee
2	Moderate	Mottling does not exceed the superior edge of the kneecap
3	Mild	Mottling does not exceed the middle thigh
4	Severe	Mottling does not exceed beyond the fold of the groin
5	Grave	Mottling exceeds beyond the fold of the groin

Adapted from Ait–Oufella et al.^17^

Skin temperature gradients, the difference between two different measurement points, such as between the forearm and fingertip or central core to the toe, can be useful in detecting changes in skin perfusions in sepsis and septic shock.^20,21^ The advantage of using skin temperature gradients between, for example, the forearm and fingertip, instead of a single skin temperature, is that both spots are similarly affected by ambient temperature. The normal skin temperature gradient between the forearm and fingertip is 0°C. Skin temperature gradients between the forearm and fingertip >4°C were associated with severe vasoconstriction. Increased skin temperature gradient was related to the outcome of sepsis.^22^

(2) Is the passive leg raise test feasible in resource-limited settings and can simple tools replace frequently lacking direct measurements of cardiac output?

Recommendation: We suggest using the passive leg raise test to guide fluid resuscitation in sepsis or septic shock in resource-limited settings (2A). It is uncertain whether the passive leg raise test has predictive values in all types of sepsis and septic shock, like in severe malaria or severe dengue. We suggest using the passive leg raise test in children, but only in those older than 5 y of age (2C). We recommend direct measurement of changes in cardiac output when performing a passive leg raise test (1C) and suggest using changes in pulse pressure if the former is not possible (2C).

##### Rationale

If it is decided that a patient is hypovolemic, it should also be determined whether that patient is fluid responsive. The Surviving Sepsis Campaign weakly recommends the use of dynamic vs static variables like the passive leg raise test.^1^ A search of the literature combining various search terms for ‘passive leg raise’ alone and in combination with diverse search terms covering ‘sepsis or septic shock’ and ‘resource-limited settings’ failed to identify any investigation originating from resource-limited settings.

**Figure 2.**
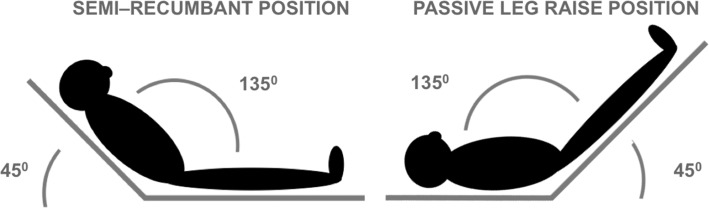
For maximum reliability, a passive leg raise test should be performed following some rules. One possible variation of the test starts from a semi-recumbent position. The second step is to raise the legs, maintaining the angle between them using the automatic motion of the bed to avoid artefacts. The third step returns the patient to the semi-recumbent position to ensure that the patient recovers the previous haemodynamic parameters.

The method for performing the passive leg raise test is important because it fundamentally affects its haemodynamic effects and reliability.^26^ The test needs to be executed so that it does not result in pain and anxiety, as this may influence the results. Furthermore, a proper passive leg raise test consists of lifting the bed at the foot end, not lifting the legs (Figure 2). The latter could be a challenge in resource-limited settings where beds are usually not easily adjustable. While it is best to use a direct measure of cardiac output or stroke volume, this is frequently impossible in settings in low-resource settings. A less accurate but still acceptable approach is to detect changes in pulse pressure. The test starts with an initial (non-invasive) blood pressure measurement and after 60–90 s of passively raising the legs the blood pressure measurement is repeated. A change in the difference between the systolic and diastolic pressure >15% could indicate that the patient is fluid responsive.^27^

It remains uncertain whether the passive leg raise test has comparable predictive values in various types of sepsis and septic shock, e.g., in severe malaria or severe dengue, as literature is lacking. This could actually be seen as one major objection against widespread use of the passive leg raise test in resource-limited settings. This is also true for young children. So far only one preliminary study suggests that a passive leg raise test is helpful in predicting fluid responsiveness in children, but not in those younger than 5 y of age.^28^

#### Recommendations for fluid strategies

(3) Which intravenous fluids should be used for fluid resuscitation in sepsis and septic shock in resource-limited ICUs?

Recommendation: We recommend crystalloid solutions as the initial fluid of choice in patients with severe bacterial sepsis or septic shock (1B) and recommend against the use of synthetic colloid solutions (1B). We recommend the same for patients with severe falciparum malaria (1B). We also recommend using crystalloids and not colloids for initial fluid resuscitation (1B) in severe dengue with compensated shock, but there is insufficient evidence to recommend fluid choices in severe dengue with hypotensive shock. In order to avoid delays in initial resuscitation, it is advisable that wards caring for patients with sepsis or septic shock stockpile crystalloid solutions for their immediate availability to avoid delaying initial fluid resuscitation (UG).

##### Rationale

There is a large body of literature from resource-rich settings on the choice of fluids in severe sepsis and septic shock, with a strong focus on sepsis caused by bacterial pathogens. The theoretical benefits of colloid solutions over crystalloids, with better retention in the intravascular compartment, has not translated to better outcomes with colloids for the treatment of severe sepsis or septic shock in randomized clinical trials performed in resource-rich settings. In addition, synthetic colloid solutions have shown important adverse effects, in particular nephrotoxicity with the use of starch solutions. Consequently the Surviving Sepsis Campaign makes a strong recommendation for the use of crystalloid solutions over colloids for fluid resuscitation.^1^ A search for evidence originating from resource-limited settings and for specific causes of sepsis or septic shock in these settings, like malaria and dengue, resulted in seven additional articles.^29–35^

The Fluid Expansion As Supportive Therapy (FEAST) trial in children in sub-Saharan Africa with compensated septic shock, of which 57% had severe falciparum malaria, showed a detrimental effect of saline bolus as well as albumin bolus therapy compared with a more conservative fluid therapy.^29^ The study supersedes earlier small studies suggesting a survival benefit of albumin infusion over crystalloids in children with severe falciparum malaria and severe sepsis.^30,31^

Three randomized trials in patients with dengue shock syndrome did not show better outcome parameters with (more expensive) colloids vs crystalloid fluids.^32–34^ A quasi-randomized study from the Philippines alternating the allocation of colloids with crystalloids also did not show an additional benefit of colloids.^35^

From the task force members’ experience, it is important that in wards caring for critically ill patients, intravenous fluids should be stockpiled so that they are immediately available for emergency treatment, to save time and to prevent incurring additional costs for the patient’s family.

(4) How much and how fast should fluids be administered intravenously in sepsis or septic shock in resource-limited ICUs?

Recommendations: We recommend that fluid resuscitation should be initiated in patients with sepsis and suspected hypovolaemia as early as possible, ideally within the first 30 min after recognition, and to start with 30 ml/kg over the first 3 h (1A). Larger amounts of fluid may be needed if the patient remains fluid responsive (e.g., according to the results of a passive leg raise test) and still shows signs of tissue hypoperfusion (e.g., according to the capillary refill time, the skin mottling score or skin temperature gradients) (1C). We recommend being extremely cautious and thus more conservative in patients in settings with no or limited access to vasopressors and mechanical ventilation, where consideration should be given to stopping fluid administration if the patient develops signs of respiratory distress or lung crepitations on chest auscultation (1A). This also applies for fluid resuscitation in children (1A).

##### Rational

A landmark study from an emergency department in a resource-rich setting found that so-called early goal-directed therapy, in which intravenous fluids were given to swiftly return physiological parameters to predefined levels, reduced mortality by as much as one-third.^36^ Early goal-directed therapy has since become mainstream practice in the treatment of critically ill patients. The Surviving Sepsis Campaign recommends that in the resuscitation from sepsis-induced hypoperfusion, at least 30 ml/kg of intravenous crystalloid fluid be given within the first 3 h.^1^ A systematic search of the literature was performed combining the search terms ‘goal-directed therapy’ with ‘sepsis’ or ‘infection’ and ‘resource-limited settings’, yielding five additional articles originating from resource-limited settings.^29,37–40^

The largest fluid trial performed in resource-limited settings is the above-cited FEAST trial in children.^29^ This trial showed an alarming increase in mortality with bolus intravenous infusion in critically ill children. There is an ongoing debate about whether mortality increased because of the development of pulmonary fluid overload that could not be compensated for by mechanical ventilation. A secondary analysis of FEAST exploring whether boluses may have caused excess deaths from fluid overload actually suggested cardiovascular collapse rather than fluid overload appeared to contribute most to excess deaths with rapid fluid resuscitation.^41^ Nevertheless, similar alarming findings come from several studies in adult patients in resource-limited settings.^37–40^ The most recent trial clearly showed a protocol for early resuscitation with administration of intravenous fluids and vasopressors increased mortality.^40^ The absolute or relative absence of vasopressors, and maybe mechanical ventilation, could make fluid loading too dangerous.

#### Recommendations for vasopressors and inotropes

(5) What is the best choice, timing and method of administration of vasopressors and inotropes in sepsis and septic shock in resource-limited settings?

Recommendation: We recommend against the start of a vasopressor before initial fluid resuscitation, especially when a central line cannot be used (1C). We suggest starting a vasopressor in patients with persistent arterial hypotension (2C) and recommend targeting a mean arterial blood pressure ≥65 mmHg (1B). We recommend using norepinephrine (noradrenaline) as a first-line vasopressor (1B) and suggest using dopamine if norepinephrine is not available (2B). The target for titration of inotropic drugs could be normalization of plasma lactate levels (<2 mmol/L), normalization of capillary refill time (<3 s) or reduction in skin mottling (UG) if plasma lactate levels cannot be measured. We suggest using dobutamine as a first-line inotrope (2B) and epinephrine (adrenaline) if dobutamine is not available (2B). We recommend administering vasopressors via a central venous line (1C) and suggest titrations of vasopressors and inotropes using a syringe or infusion pump when available (2D).

##### Rational

The Surviving Sepsis Campaign recommends norepinephrine as the first-choice vasopressor and adding epinephrine to norepinephrine with the intent of raising mean arterial pressure to target to decrease norepinephrine dosage. The Surviving Sepsis Campaign also suggests using dopamine as an alternative vasopressor only in selected patients and using dobutamine in patients who show evidence of persistent hypoperfusion despite adequate fluid loading and the use of vasopressors.^1^ A systematic search of the literature combining search terms ‘vasopressors’, ‘catecholamines’ and ‘inotropes’ with ‘sepsis or septic shock’ and ‘resource-limited settings’ yielded only two relevant articles originating from resource-limited settings.^42,43^ We largely follow the recommendations of the Surviving Sepsis Campaign,^1^ but provide additional recommendations mainly based on task force members’ experiences.

Extravasation of vasopressors can cause skin necrosis and extravasation is more likely with administration through a peripheral infusion line compared with central venous administration. Central venous catheters, however, are frequently not available, expensive (sometimes requiring extra payments from the patient or family members) and inserted too late. Administration of vasopressors is thus frequently done through a peripheral line. We consider it reasonable to await the effect of initial fluid resuscitation before starting infusion of vasopressors through a peripheral infusion line, but in patients with extremely low blood pressure, and in those not immediately responding to initial fluid loading, it may be necessary to continue without a central venous catheter. Additional advantages of a central venous line are that it can also be used for repeated blood sampling, measurement of static haemodynamic measures and, where possible, follow-up of central venous oxygenation.

Vasopressors and inotropes have a narrow therapeutic window, necessitating accurate dosing. Continuous administration at exact doses is safeguarded preferably by automatic infusion with a syringe or infusion pump. Although less accurate, when syringe pumps are not available, these drugs can be diluted in normal saline and administered using a mechanical drop counter.

Norepinephrine is not generally available in hospitals with limited resources. Dopamine is more widely available, but reported best access in resource-limited settings is to epinephrine. We prefer dopamine to epinephrine, as epinephrine may cause lactate acidosis.^42,43^ In resource-limited settings, dobutamine is only available in selected regions, and stock outages of the drug are very common.

Titration of inotropes in resource-limited ICUs is a challenge, as assessed by means of plasma lactate levels is expensive, and is frequently not possible. Capillary refill time (<3 s) and the skin mottling score can be used to evaluate the effect of infusion of vasopressors and inotropes, but there is no documented evidence regarding efficacy or safety. And it should be noted that vasopressors can affect capillary refill time and skin mottling scores.

## Conclusions

An international team of six physicians from resource-rich and -limited settings reported on a set of pragmatic recommendations for haemodynamic assessment and support in patients with sepsis and septic shock in resource-limited settings. The paucity of evidence from resource-limited settings and in specific types of sepsis and septic shock underscores the urgent need for rigorous trials, since efficacy and effectiveness of commonly used interventions in resource-rich settings can differ greatly in resource-limited settings.

## Supplementary data

Supplementary data are available at Transactions online (http://trstmh.oxfordjournals.org/).

## Supplementary Material

Supplementary Data
