# Supplementary material for: Haemodynamic assessment and support in sepsis and septic shock in resource-limited settings
Source: Trans R Soc Trop Med Hyg. 2018 Feb 9;111(11):483–9. doi: 10.1093/trstmh/try007 (PMC5914406; doi:10.1093/trstmh/try007)
Supplement: Supplementary Data [file supplementtotrstmh-d-17-00191revised.docx]

Supplement to:

**Hemodynamic Assessment and Support in Sepsis and Septic Shock in Resource–limited Settings**

David Misango^1^, Rajyabardhan Pattnaik^2^, Tim Baker^3,4^, Martin W. Dünser^5^, Arjen M. Dondorp^6,7,8^ and Marcus J. Schultz^6,8^, for the ‘European Society of Intensive Care Medicine (ESICM) Global Intensive Care’ working group* and the ‘Mahidol–Oxford Research Unit’ (MORU) in Bangkok, Thailand

**Aga Khan University Hospital, Nairobi, Kenya:**

^1^Department of Anaesthesiology and Critical Care Medicine

**Ispat General Hospital, Rourkela, Sundargarh, Odisha, India:**

^2^Department of Intensive Care Medicine

**Karolinska University Hospital, Stockholm, Sweden:**

^3^Department of Anesthesia, Intensive Care and Surgical Services

**Karolinska Institutet:**

^4^Department of Public Health Sciences

**Salzburg General Hospital, Salzburg, Austria:**

^5^Department of Anesthesiology, Perioperative and General Critical Care Medicine

**Faculty of Tropical Medicine, Mahidol University, Bangkok, Thailand:**

^6^Mahidol–Oxford Tropical Medicine Research Unit (MORU)

Nu**ffield Department of Clinical Medicine, University of Oxford, Oxford, UK:**

^7^Oxford Centre for Tropical Medicine and Global Health

**Academic Medical Center, University of Amsterdam, Amsterdam, The Netherlands:**

^8^Department of Intensive Care

*Taskforce members involved in the development of this set of

recommendations are shown on the next page

**Correspondence**

Marcus J. Schultz

Mahidol–Oxford Tropical Medicine Research Unit (MORU), Faculty of Tropical Medicine, Mahidol University

420/6 Rajvithi Road, Bangkok 10400, Thailand

E–mail: [marcus.j.schultz@gmail.com](mailto:marcus.j.schultz@gmail.com)

**Taskforce members**

(Heads) Arjen Dondorp (Faculty of Tropical Medicine, Mahidol University, Bangkok, Thailand & Academic Medical Center, University of Amsterdam, Amsterdam, The Netherlands &), Martin Dünser (Innsbruck Medical University, Innsbruck, Austria), and Marcus Schultz (Academic Medical Center, University of Amsterdam, Amsterdam, The Netherlands & Faculty of Tropical Medicine, Mahidol University, Bangkok, Thailand).

(Other group members) Neill K.J. Adhikari (Sunnybrook Health Sciences Centre & University of Toronto, Toronto, ON, Canada), Derek Angus (University of Pittsburgh, Pittsburgh, PA), Luciano Azevedo (Hospital Sirio–Libanes, Saõ Paulo, Brazil), Kwizera Arthur (Mulago National Referral Hospital, Kampala, Uganda), Tim Baker (Karolinska Institute, Stockholm, Sweden), Ted Barnett (The Ochsner Medical Center, New Orleans, Louisiana), Chris Farmer (Mayo Clinic, Rochester, MI), Rashan Haniffa (Faculty of Tropical Medicine, Mahidol University, Bangkok, Thailand), Randeep Jawa (Stony Brook University Medical Center, Stony Brook, NY, USA), Niranjan Kissoon (British Columbia Children’s Hospital and University of British Columbia, Vancouver, Canada), Rakesh Lodha (All India Institute of Medical Science, Delhi, India), Ganbold Lundeg (Mongolian National University of Medical Sciences, Ulaanbaatar, Mongolia), Ignacio Martin Loeches (St. James's University Hospital, Dublin, Ireland), David Misango (Aga Khan University Hospital, Nairobi, Kenya), Mervyn Mer (Johannesburg Hospital and University of the Witwatersrand, Johannesburg, South Africa), Sanjib Mohanty (Ispat General Hospital, Rourkela, Sundargarh, Odisha, India), Srinivas Murthy (BC Children’s Hospital, University of British Columbia, Vancouver, Canada), Ndidiamaka Musa (Seattle Children’s Hospital and University of Washington, WA), Jane Nakibuuka (Mulago National referral and university teaching hospital, Kampala, Uganda), Mai Nguyen Thi Hoang (Oxford University Clinical Research Unit, Ho Chi Minh City, District 5, Vietnam), Binh Nguyen Thien (Trung Vuong Hospital, Ho Chi Minh City, Viet Nam), Rajyabardhan Pattnaik (Ispat General Hospital, Rourkela, Sundargarh, Odisha, India), Luigi Pisani (University of Bari Aldo Moro, Bari, Italy), Jason Phua (Yong Loo Lin School of Medicine, National University of Singapore, Singapore, Singapore), Jacobus Preller (Addenbrooke’s Hospital, Cambridge University Hospitals NHS Foundation Trust, Cambridge, UK), Pedro Povoa (Nova Medical School, CEDOC, New University of Lisbon, Lisbon, Portugal & Hospital de São Francisco Xavier, Centro Hospitalar de Lisboa Ocidental, Lisbon, Portugal), Suchitra Ranjit (Appolo’s Hospitals, Chennai, India), Jonarthan Thevanayagam (Mzuzu Central Hospital, Mzuzu, Malawi), and Louise Thwaites (Centre for Tropical Medicine and Global Health, Nuffield Department of Medicine, University of Oxford, UK).

**Guideline development**

*Selection of taskforce members*

The selection of the taskforce members was based on interest in specific aspects of sepsis and septic shock, and hands–on experience in intensive care units (ICUs) in resource–limited settings. Marcus Schultz, Martin Dünser and Arjen Dondorp contacted potential team members through email and in person at the ‘27^th^ Annual Congress of the European Society of Intensive Care Medicine’ in Barcelona, Spain from 27 September – 1 October 2014, and the ‘35^th^ International Symposium on Intensive Care and Emergency Medicine’ in Brussels, Belgium from 17 March – 20 March 2015, and created nine subgroups assigned to nine areas in the management of sepsis and septic shock, i.e., ‘diagnosis of sepsis’, ‘levels of ICU’, ‘organizational aspects’, ‘infection control’, ‘hemodynamic support’, ‘ventilatory support’, ‘sedation, renal failure, prophylaxes, glucose control and feeding’, ‘tropical aspects’ and ‘pediatric aspects’. Additional taskforce members were appointed by the group heads to address content needs for the development process. Several taskforce members had experience in ‘Grading of Recommendations Assessment, Development and Evaluation’ (GRADE) process and use of the GRADEpro Guideline Development Tool – see supplement tables 1 and 2.

David Misango, Rajyabardhan Pattnaik, and Tim Baker were appointed as the taskforce subheads; Martin Dünser, Arjen Dondorp, and Marcus Schultz were assigned to this subteam based on their specific expertise and interest in hemodynamic assessment, fluid management and treatment with vasopressors and inotropes in resource–limited settings.

*Meetings*

An initial Internet meeting was held to establish the procedures for literature review and drafting of tables for evidence analysis. The taskforce subheads continued work via email, though several meetings occurred at major international meetings, and via teleconferences and electronic–based discussions among the subheads and with members of other subgroups.

In the first meetings, ten clearly defined questions regarding hemodynamic monitoring, fluid management, and vasoactive treatment were formulated using the GRADEpro Guideline Development Tools. These ten questions were extensively evaluated for content and clarity amongst the subgroup members and were reduced to five questions to prevent overlap with other subgroups. After approval by the subgroup members, the subgroup heads split up, each one to seek for evidence for recommendations regarding two or three of the specific questions posed, seeking help from other members in identifying relevant publications where necessary. The heads summarized the evidence and formulated the recommendations after multiple discussions via email. These were communicated among the all members. After their approval, the subheads summarized the evidence in a report, which was sent for approval by all members of the taskforce.

*Search techniques*

The search for literature followed the same techniques as described for the development of the ‘Surviving Sepsis Campaign: International Guidelines for Management of Sepsis and Septic Shock: 2016’, in short the ‘Surviving Sepsis Campaign’–guidelines’ [1]. In case a question was identical to one in those guidelines, the subgroup members only searched for additional articles, specifically (new) metaanalyses related to the questions, in a minimum of one general database (i.e. PubMed, MEDLINE, EMBASE) and the Cochrane Libraries. Furthermore, the subgroup members attempted to identify running investigations in resource–limited settings.

*Grading of recommendations*

The taskforce members followed the principles of GRADE. In short, GRADE classifies quality of evidence as high (Grade A), moderate (Grade B), low (Grade C), or very low (Grade D) and recommendations as strong (Grade 1) or weak (Grade 2). The factors influencing this classification are presented in the Supplement tables 1 and 2. The subgroup members paid extensive attention to several relevant factors for settings were resources are limited, including aspects like availability and feasibility, and affordability and safety in resource–limited settings. A strong recommendation was worded as ‘*we recommend*’ and a weak recommendation as ‘*we suggest*’. A number of recommendations could remain ‘ungraded’ (UG), when, in the opinion of the subgroup members, such recommendations were not conducive for the process described above.

*Conflicts of interest*

None of the taskforce members represented industries, and there was no industry input into the development of this report. None of the members received honoraria for any role in the process. Each member provided a standard COI–form, to be uploaded through the GRADEpro Guideline Development Tool website. Also, none of the members reported any conflicts of interest.

**TABLES**

| **Supplement Table 1.** Quality of Evidence | | |
| --- | --- | --- |
| A | Randomized controlled trials | High |
| B | Less well performed randomized controlled trial(s) or well performed observational studies | Moderate |
| C | Observational studies | Low |
| D | Less well performed observational studies or expert opinion | Very low |
| Factors that may decrease the strength of evidence: poor quality of planning and implementation of available RCTs, suggesting high likelihood of bias; inconsistency of results, including problems with subgroup analyses; indirectness of evidence (differing population, intervention, control, outcomes, comparison); imprecision of results; and high likelihood of reporting bias.  Factors that may increase the strength of evidence: large magnitude of effect (direct evidence, relative risk > 2 with no plausible confounders); very large magnitude of effect with relative risk > 5 and no threats to validity (by two levels); and dose–response gradient. | | |

| **Supplement Table 2. Strong versus Weak Recommendations*** | |
| --- | --- |
| *What Should be Considered* | *Recommended Process* |
| High or moderate evidence | The higher the quality of evidence, the more likely a strong recommendation. |
| Certainty about the balance of benefits vs. harms and burdens | The larger/smaller the difference between the desirable and undesirable consequences and the certainty around that difference, the more likely a strong/weak recommendation. |
| Certainty in or similar values | The more certainty or similarity in values and preferences, the more likely a strong recommendation. |
| Resource implications | The lower/higher the cost of an intervention compared to the alternative the more likely a strong/weak recommendation. |
| Availability and feasibility in resource–limited settings | The less available, the more likely a weak recommendation |
| Affordability for resource–limited settings | The less affordable, the more likely a weak recommendation |
| Safety of the intervention in resource–limited settings | The less safe in a resource–limited settings, the more likely a weak recommendation |
| *, in case of a strong recommendation we use ‘we *recommend* …’; in case of a weak recommendation we use ‘we *suggest* …’ | |
